# Are healthy smokers really healthy?

**DOI:** 10.1186/s12971-016-0101-z

**Published:** 2016-11-15

**Authors:** Zijing Zhou, Ping Chen, Hong Peng

**Affiliations:** Department of Respiratory Medicine, the Second Xiangya Hospital, Central South University, 139 Renmin Middle Road, Changsha, Hunan 410011 People’s Republic of China

**Keywords:** Healthy smokers, Respiratory system, Inflammation, Immune changes, Genetic alterations

## Abstract

Cigarette smoke contains more than 4500 chemicals which have toxic, mutagenic and carcinogenic effects. Strong evidences have shown that current smokers take a significantly higher risk of cardiovascular diseases, chronic obstructive pulmonary disease (COPD) and lung cancer than nonsmokers. However, less attention has been paid to the smoking induced abnormalities in the individuals defined as healthy smokers who are normal with spirometry, radiographic images, routine physical exam and categorized as healthy control group in many researches. Actually, ‘healthy smokers’ are not healthy. This narrative review focuses on the smoking related pathophysiologic changes mainly in the respiratory system of healthy smokers, including inflammation and immune changes, genetic alterations, structural changes and pulmonary dysfunction.

## Background

Cigarette smoke is a multipotent mixture of thousands of components that have toxic, mutagenic and carcinogenic properties. Numerous chemicals are added to the tobacco content, paper, and filter during the manufacturing process. Among the mainstream smoke emissions from cigarettes, polycyclic aromatic hydrocarbons, N-Nitrosamines, nickel, cadmium, chromiun, arsenic, misc organic compounds are known carcinogens. Nicotine, carbon monoxide, acrolein and reactive oxidant substances are toxins that can cause immune dysfunction. Moreover, nicotine is the predominant addictive cigarette smoke constituent [1]. Epidemiological studies demonstrate that smoking is a significant risk factor for cardiovascular diseases [2], chronic obstructive pulmonary disease (COPD) [3] and lung cancer [4]. Thus, much work has been done on the molecular and cellular abnormalities in those patients who have a smoking history with smoking related diseases, trying to find the differences in comparison with healthy nonsmokers and the possible mechanisms. However, until recently, there has not been much focus on the individuals categorized as healthy smokers who are asymptomatic with normal spirometry, radiographic images and physical exam, appearing to be much healthier than those smokers who have already developed diseases [5]. Interestingly, when compared with subjects who never smoked, the point that asymptomatic smokers with normal physical exam are healthy is much less obvious. Actually, healthy smokers are not healthy. Many studies have detected pathological changes in healthy smokers. In the present paper, we will mainly review the pathophysiologic changes in the respiratory system of smokers with normal physical exam, including inflammation and immune changes, genetic alterations, structural changes and pulmonary dysfunction.

## Inflammation and immune changes

The analysis of the induced sputum, expired breath condensate (EBC), bronchoalveolar lavage (BAL) and biopsies of lung tissue from ‘healthy smokers’ show strong evidences of inflammatory and immune changes in the airway.

### Induced sputum

The presence of neutrophils in sputum is one of the most common landmarks of inflammatory changes brought by smoking. As early as 1992, Swan et al. [6] found smokers with a greater number of pack years tended to have significantly higher levels of all cytomorphologic components, including neutrophils. Moreover, the neutrophil rating declined over the follow-up in quitters, while it increased among non-quitters. In fact, lots of researches have demonstrated elevated level of both the percentage and absolute number of neutrophils in induced sputum from healthy smokers as compared with nonsmokers [7–9].

The phenotype of macrophages in induced sputum is definitely altered by smoking. Domagala- Kulawikan et al. [10] detected increased expression of CD54 and CD71, which had effects on the metabolic activity of macrophages, in induced sputum of smokers compared with nonsmokers. CD54 is an adhesion molecule that mediates adhesion and the cell-cell interactions of macrophages, while CD71 has the function of proliferation and maturation [10, 11]. The up-regulation of CD54 and CD71 indicate Besides, the proportion of CD14 positive macrophages in induced sputum, which has been proved to play a role in macrophage activation in infection and inflammatory processes in COPD, was higher in smokers than nonsmokers [10, 12].

Biochemical analysis also showed a lower percentage of CD8^+^T lymphocytes and a higher ratio of CD4^+^/CD8^+^ T-cells in induced sputum of healthy smokers as compared with that of nonsmokers. After 6 months-cessation, the percentage of CD8^+^ T-cells increased in quitters and a ratio of CD4^+^/CD8^+^ decreased [13]. As the major activity ofCD8^+^ T lymphocytes is the facilitation of the rapid resolution of acute viral infections, the lower percentage of CD8^+^ T-cells suggests that smokers may have a deficit in cell-mediated immunity in the lung and may explain the increased susceptibility of smokers for viral infections. The alteration of other chemicals or cytokines that represents inflammatory and immune processes in healthy smokers were also found in their induced sputum. Takanashi with his team [14] observed a significant reduction in IL-10 levels and a small number of IL-10-expressing cells in the sputum of patients with asthma and COPD and healthy smokers compared with nonsmokers. The decreased level of IL-10, an anti-inflammatory cytokine with major down-regulatory effects on inflammation, may contribute to the development of chroni cairway inflammation among smokers. Both CCL5 and CCR1 were up-regulated on inflammatory cells of induced sputum of healthy smokers compared with nonsmokers [15, 16]. According to one of the latest studies, the level of IL-6, IL-8 and tumor necrosis factor alpha (TNF-α) which were positively correlated with smoking load (pack-years) in induced sputum of healthy smokers were higher than that of nonsmokers [17]. All the three cytokines are important markers of inflammation and play key roles in the persistence of inflammatory process in COPD [18].

### Expired breath condensate (EBC)

Much attention has been paid to the changes brought by smoking in smokers’ exhaled breath condensate (EBC). Many studies have found lower pH values in EBC, as reflected airway inflammation, in diverse inflammatory airway diseases, including bronchial asthma, bronchiectasis and COPD [19]. In healthy smokers, mean PH values lower than those observed in healthy non-smokers have always been reported. The ECLIPSE study found that EBC PH was significantly reduced both in COPD patients and chronic healthy smokers compared to healthy non-smokers [20], but there were no differences between COPD patients and healthy smokers. This result is consistent with Koczulla’s [21] and Nicola’s study [22]. Since acidification of the airways reflects airway inflammation, the lower PH value in healthy smokers’ EBC suggest the inflammatory changes in their airways.

Chronic smoking also alters the level of inflammatory markers in EBC of smokers who remain symptomless and seem to be healthy on the surface. Elevated concentrations of IL-6 in EBC, a pro-inflammatory cytokine produced by epithelial cells and macrophages in the airways, was observed in healthy smokers compared to nonsmokers [23]. Higher concentrations of leukotriene (LT)B4, another marker of inflammation, was also detected in EBC of both COPD patients and healthy smokers than in nonsmokers [23]. Garey with his team [24] demonstrated that neutrophil chemotactic activity were significantly higher in EBC of smokers in comparison to non-smokers. This observation was reconfirmed by Corhay after three years and it was in keeping with the fact that neutrophils were well known to be increased in the airways of smokers [25]. Besides, smokers also showed higher TNF-α levels in EBC [26].

In recent years, evidence has emerged that oxidative stress plays a crucial role in the development and perpetuation of inflammation. Higher 8-isoprostane and H_2_O_2_ levels in EBC of subjects with COPD and smokers than non-smokers have been reported [27]. Isoprostanes are produced by ROS mediated peroxidation of arachidonic acid. The oxidative stress brought by smoking also promotes the inflammatory process.

### Bronchoalveolar lavage (BAL)

The first paper detailing BAL dealt with normal values was published in 1974 [28]. Over the following years, BAL has been used to investigate inflammatory and immune processes in the lower respiratory tract which is able to give us a deeper understanding of pathophysiologic changes brought by smoking.

Typically smokers have a decrease of CD4^+^/CD8^+^ caused by higher percentage of CD8^+^ T-cells in BAL as compared with nonsmokers [29]. The same change of T-lymphocyte subsets was also demonstrated in the lung tissue of healthy smokers, but was in contrast with the result detected in induced sputum of smokers that decreased proportion of CD8^+^T lymphocytes with increased ratio of CD4^+^/CD8^+^ T-cells. One explanation would be the inflammatory microenvironment in airway lumen sampled by induced sputum is different from that in the BAL and airway epithelium sampled by bronchial biopsies [30]. Another reason would be smoking induced suppression of the trans-epithelial migration of CD8^+^ lymphocytes, increasing their number in the large airway wall, while reducing their number in the airway lumen [31]. Besides, among the subsets of CD8^+^T-lymphocytes, Yu et al. [32] showed a significant trend for greater Tc1/Tc2 ratio in BAL of patients with COPD and smokers compared with nonsmokers. CD8^+^T-lymphocytes who are key inflammatory effector and regulatory cells have been proved to play an important role in the inflammatory process of COPD [33]. CD8^+^T-lymphocytes can be differentiated into cells that synthesize interferon-gamma (IFN-γ) but not interleukin-4 (IL-4) (Tc1 cells) or cells that synthesize IL-4 but not IFN-γ (Tc2 cells) [34]. However, little is known about which subpopulation is mostly involved in the immuno-pathogenesis of COPD. The imbalance of the two phenotypes was actually detected in the BAL of smokers and patients with COPD.

Kuschner with his co-workers [35] observed greater concentrations of monocyte chemoattractant protein (MCP)-1 with increased level of IL-6, IL-8 and IL-1β in BAL of control smokers as compared with nonsmokers, moreover, the level of IL-8 and IL-1β were elevated in a cigarette dose-dependent manner. Clara cell 10 kDa protein (CC10), which may have a role in protecting the respiratory tract from oxidative stress and inflammation by inhibiting the expression and/or activity of proteins, such as phospholipase A2, IFN-γ, and TNF-a was found to be significantly decreased in BAL fluids of healthy smokers in comparison with nonsmokers [36, 37]. Hence, a decrease of CC10levels in the peripheral airways as a result of smoking may be associated with enhanced pro-inflammatory process in the peripheral airways of the smokers. Molecules mediating tissue damage as matrix metalloproteinase (MMP)-9 and MMP-12 had either increased levels and/or enhanced activities in samples from BAL of smokers as compared with nonsmokers [38, 39]. Surfactant protein A and D (SP-A, SP-D), members of the collectin family which play a key role in innate immunity in animal models [40], were decreased in BAL of healthy smokers vs. non-smokers [41]. Therefore, lower levels of SP-D caused by cigarette smoking may weaken lung immunity in healthy smokers.

Alveolar macrophages (AM) are responsible for a broad set of host defense functions including recognition and phagocytosis of pathogenic material and apoptotic cells. Various changes of smokers' alveolar macrophages have been noted in several studies. The number and proportion of AM in healthy smokers’ BAL are increased as compared with nonsmokers [42, 43]. And AM from smokers differ from those of nonsmokers in that they are slightly larger, and contain more golgi vesicles, endoplasmic reticulumand residual bodies which contain distinctive fiber-like inclusions [44, 45]. Besides the ultrastructural alterations, the function of AM is also changed. Compared with nonsmokers, alveolar macrophages of cigarette smokers has a significantly greater esterase and protease activity with higher resting metabolism and enhanced lysozyme secretion [44, 46, 47]. However, studies showed AM of smokers had impaired phagocytic capability [48]. More interestingly, in contrast with elevated level of IL-6, IL-8 and IL-1β detected in BAL of healthy smokers in comparison with nonsmokers, the decreased capacity of smokers’ AM to release IL-1, IL-6, IL-8 and TNF-α has been oberserved in many studies and this decreased secretion of cytokines may result in impairment of pulmonary immune responses in smokers with increased incidence of infection [49–52]. One possible explanation would be that these cytokines are produced not only by AM but other cells in BAL. Another reason would be the increased number of AM in BAL which lead to the impaired cytokine secretion by smokers’ AM appearing to be offset. Furthermore, smokers’ AM produces significantly more superoxide anions that may contribute to the lung injury [53, 54]. Cigarette smoke can also change the phenotype of AM. Schaberg T et al. [55] found that much more AM from smokers expressed CD11a, CD11b, CD11c and CD18 as compared with nonsmokers. AM of both healthy smokers and patients with COPD exhibited a unique polarization pattern which was different from nonsmokers’. The analysis from Shaykhiev et al. [56] revealed that M1 polarization related genes which are relevant to inflammation and cell-mediated immunity were down-regulated in AM of smokers and COPD individuals with a smoking history, while M2 related genes closely associated with anti-inflammatory cytokines and molecules implicated in tissue remodeling were up-regulated. Therefore, the result from Shaykhiev et al.’s study is consistent with the previous finding that decreased capacity of smokers’ AM to release pro-inflammatory cytokines, suggesting AM may contribute to smoking related diseases in a non-inflammatory manner.

### Biopsies of lung tissue

Histopathological examinations help us find inflammatory alterations in bronchial biopsies of smokers without any symptoms, including vascular hyperplasia, submucosal edema, inflammatory cell infiltrates and goblet cell hyperplasia [57]. An abnormal cellular infiltrate into the airway submucosa of smokers is always reported. Lams et al. [58] found an increase in small-airway neutrophils, total eosinophils and a trend toward an increase in CD 8 + cells in smokers as compared to nonsmokers. Two studies from European countries confirmed a larger number of CD3+, CD8+, CD68+ cells in the bronchial submucosa of smokers compared with nonsmokers [59, 60]. Isajevs et al. [61] demonstrated again a higher level of neutrophils, macrophages and CD8 + cells both in large and small airways of smokers than nonsmokers, but lower than that of subjects with COPD which is consistent with Saetta’s finding [33] that smokers who developed symptoms of chronic bronchitis and chronic airflow limitation had an increased number of CD8+ cells in the peripheral airways as compared with asymptomatic smokers with normal lung function, suggesting this inflammatory process may be under control. Nuclear factor-kB (NF-kB), a transcription factor regulating the expression of many genes involved in inflammation [62], is increased in airways of asymptomatic smokers as compared with nonsmokers [61, 63]. Besides, the expression of p65 NF-kB, one of its activated form, is also elevated in the epithelium of smokers with normal lung function and COPD patients that correlated with a greater counts of macrophages, neutrophilic leucocytes and CD8+ T cells in airway walls, when compared to nonsmoking persons [61, 63]. The level of CXCL6 and its receptor, CXCR1 which can induce leukocyte recruitment and activation at sites of inflammation [64] are increased in the epithelium and submucosa of healthy smokers respectively [65].

Moreover, Wang with his team [66] demonstrated the toll-like receptor(TLR)5, expressed mainly on the apical side of the epithelium, was down-regulated in healthy smokers and smokers with COPD, compared to nonsmokers. The toll-like receptors are important components of the respiratory epithelium host innate defense and TLR-deficient mice develop exhibit impaired CD4^+^T cell response to a flagellated pathogen [67], suggesting suppression of airway epithelial TLR5 may contribute to the increased susceptibility of smokers and smokers with COPD to airway flagellated bacterial infection.

## Genetic alterations

Epidemiological data has shown that long-term smokers are taking a greater risk of developing COPD and lung cancer as compared with nonsmokers. One of the possible reasons is the smoking induced genetic alterations which modify susceptibility to lung diseases. For smokers, those up- or down-regulation of gene expressions with relevant impaired biological function accelerate the progress of respiratory disorders.

### Genetic alterations in alveolar macrophages

Human alveolar macrophages, mostly residing on the respiratory epithelial surface, are critical components of the innate immune system. The gene expression of alveolar macrophages has been altered in active smokers when compared with nonsmokers. Table 1 shows up- or down- regulated genes (>2.0 fold change) in AM of smokers reported by at least two different studies [68–72]. The MMPs comprise a family of at least 20 proteolytic enzymes that play an essential role in tissue remodeling. Several studies in animals and humans have provided evidence that MMP12 (human macrophage elastase) is important in airway inflammation and the development of emphysema. For instance, MMP12-knockout mice exposed to cigarette smoke do not develop emphysema [73]. MMP12 up-regulation is also demonstrated to play a critical role in emphysema to lung cancer transition that is facilitated by inflammation [74]. CYP1B1, a member of the P450 superfamily with high affinity for inhaled tobacco carcinogens, is commonly expressed in human lung [75]. Lao et al. [76] found that CYP1B1 Leu432Val polymorphism acted as a risk factor for the carcinogenesis of lung cancer.

**Table 1:** Up- and down- regulated genes (>2.0 fold change) in alveolar macrophages of ‘healthy smokers’

Gene symbol	Description	Regulation (HS^a^/NS^b^)	Reference
PLA2G7	phospholipase A2, group VII	up	Woodruff et al. [68], Graff et al. [69], Philibert et al. [70]
SPP1	secreted phosphoprotein 1 (osteopontin)	up	Woodruff et al. [68], Graff et al. [69]
CYP1B1	cytochrome P450, family 1, subfamily B, polypeptide 1	up	Woodruff et al. [68], Graff et al. [69], Philibert et al. [70]
ATP6VOD2	ATPase, H+ transporting, lysosomal 38 kDa, V0 subunit d2	up	Woodruff et al. [68], Graff et al. [69]
SLC7A11	solute carrier family 7, member 11 (xCT)	up	Woodruff et al. [68], Graff et al. [69]
MMP12	matrix metallopeptidase 12 (macrophage elastase)	up	Woodruff et al. [68], Graff et al. [69] Heguy et al. [71]
FABP3	fatty acid binding protein 3	up	Woodruff et al. [68], Graff et al. [69]
FLT1	fms-related tyrosine kinase 1 (VEGFR)	up	Woodruff et al. [68], Graff et al. [69], Philibert et al. [70]
A2M	alpha-2-macroglobulin	up	Woodruff et al. [68], Graff et al. [69] Heguy et al. [71]
UCHL1	ubiquitin carboxyl-terminal esterase L1	up	Woodruff et al. [68], Graff et al. [69]
S100B	S100 calcium binding protein B	up	Woodruff et al. [68], Graff et al. [69], Philibert et al. [70]
CA2	carbonic anhydrase II	up	Woodruff et al. [68], Graff et al. [69]
SLC16A6	solute carrier family 16, member 6 (monocarboxylic acidtransporter)	up	Woodruff et al. [68], Graff et al. [69]
SSBP3	single stranded DNA binding protein 3	up	Woodruff et al. [68], Graff et al. [69]
TDRD9	tudor domain containing 9	up	Woodruff et al. [68], Graff et al. [69]
C4orf18	chromosome 4 open reading frame 18 (DKFZp434L142)	up	Woodruff et al. [68], Graff et al. [69]
DNASE2B	deoxyribonuclease II beta	up	Woodruff et al. [68], Graff et al. [69]
SDC2	syndecan 2	up	Woodruff et al. [68], Graff et al. [69]
MGST1	microsomal glutathione S-transferase 1	up	Woodruff et al. [68], Graff et al. [69]
AGPAT9	1-acylglycerol-3-phosphateO-acyltransferase 9	up	Woodruff et al. [68], Graff et al. [69]
TMTSF4	transmembrane 7 superfamily member 4 (DCSTAMP)	up	Woodruff et al. [68], Graff et al. [69]
LIPA	lipase A, lysosomal acid, cholesterol esterase	up	Woodruff et al. [68], Graff et al. [69]
CSF1	Colony-stimulating factor 1	up	Heguy et al. [71], Rose et al. [72]
CCR5	Chemokine (C-C motif) receptor 5	up	Woodruff et al. [68], Graff et al. [69], Philibert et al. [70]
CXCL11	chemokine (C-X-C motif) ligand 11	down	Woodruff et al. [68], Graff et al. [69]
CXCL9	chemokine (C-X-C motif) ligand 9	down	Woodruff et al. [68], Graff et al. [69], Philibert et al. [70]
SLC19A3	solute carrier family 19 (thiamine transporter)	down	Woodruff et al. [68], Graff et al. [69]
EMR1	egf-like module containing, mucin-like, hormonereceptor-like 1 (F4/80)	down	Woodruff et al. [68], Graff et al. [69]
CXCL10	chemokine (C-X-C motif) ligand 10	down	Woodruff et al. [68], Graff et al. [69]
PDGFD	platelet derived growth factor D	down	Woodruff et al. [68], Graff et al. [69]
IGF1	insulin-like growth factor 1	down	Woodruff et al. [68], Graff et al. [69]
GBPS	guanylate binding protein 5	down	Woodruff et al. [68], Graff et al. [69]
C8B	complement component 8, beta	down	Woodruff et al. [68], Graff et al. [69]
CD69	CD69 molecule	down	Woodruff et al. [68], Graff et al. [69]
WDR69	WD repeat domain 49	down	Woodruff et al. [68], Graff et al. [69]
TNFSF10	tumor necrosis factor (ligand) superfamily, member 10 (TRAIL)	down	Woodruff et al. [68], Graff et al. [69]
IFI27	interferon, alpha-inducible protein 27 (ISG12)	down	Woodruff et al. [68], Graff et al. [69]
TRHDE	thyrotropin-releasing hormone degrading enzyme	down	Woodruff et al. [68], Graff et al. [69]
MYB	v-myb myeloblastosis viral oncogene homolog	down	Woodruff et al. [68], Graff et al. [69], Philibert et al. [70]
ARHGAP24	Rho GTPase activating protein 24	down	Woodruff et al. [68], Graff et al. [69]
TRPC6	transient receptor potential cation channel,subfamily C, member 6	down	Woodruff et al. [68], Graff et al. [69]
ITHIHS	inter-alpha (globulin) inhibitor H5	down	Woodruff et al. [68], Graff et al. [69]

^a^HS: healthy smokers, ^b^NS: nonsmokers

### Genetic alterations in airway epithelium

Airway epithelium, lined by a variety of specialized epithelial cells, represents the first point of contact for cigarette smoke. It not only plays a central role in the barrier function of airway tract, but also responds to environment-induced damage through the release of pro-inflammatory cytokines and chemokines [77]. Genes in functional categories are detected to expressed differentially in the airway epithelium in nonsmokers and smokers. Table 2 displays up- or down- regulated genes in airway epithelium of smokers reported by more than one study [78–83]. Up-regulation of antioxidant-related genes in the airway epithelium of smokers are always reported [78–83], including the glutathione pathway genes (G6pd, GCLC, GPx2, GSR, NQO1), the redox balance genes(ADH7, AKR1b1, AKR1C1, AKR1C2, AKR1C3), the pentosephosphate cycle genes(PGD, TALDO1) and the xenobiotic metabolism genes(CYP1B1). Although catalase and the superoxide dismutase (SOD) contribute a lot to antioxidative defense, the available data suggests that gene expression of catalase and SOD do not differ in the airway epithelium of smokers and nonsmokers [78, 79]. Smoking induced down-regulation of intraflagellar transport gene and cilia-related genes in the airway epithelium of healthy smokers is associated with shorter cilia which affect mucociliary clearance [84, 85]. Healthy smokers have more active MUC5AC-core gene expression compared to the nonsmokers [86]. MUC5AC is one of the major secretory mucins expressed by surface airway epithelial cells. The activated MUC5AC-core gene expression in smokers may lead to mucus hypersecretion. Down regulation of TLR5 and physiological apical junctional complex(AJC) gene in healthy smokers may be involved in smoking-related susceptibility to airway infection [66, 87]. Overexpression of ubiquitin carboxyl-terminal hydrolase L1 (UCHL1) which is used as a marker of lung cancer in chronic smokers may represent an early event in the complex transformation from normal epithelium to overt malignancy [88]. Reduced expression of Notch pathway in both smokers and patients with COPD may be responsible for the abnormal differentiation of the airways [89].

**Table 2:** Up- and down- regulated genes in airway epithelium of ‘healthy smokers’ reported

Epithelium	Gene symbol	Description	Regulation (HS^a^/NS^b^)	Reference
SAE^c^/LAE^d^	G6pd	glucose-6-phosphatedehydrogenase	up	Carolan et al. [78], Hackett et al. [79]
SAE/LAE	GCLC	Glutamate-cysteineligase, catalytic subunit	up	Carolan et al. [78], Hackett et al. [79], Spira et al. [82]
SAE/LAE	GPx2	Glutathioneperoxidase 2	up	Carolan et al. [78], Hackett et al. [79], Harvey et al. [80], Turetz et al. [81], Spira et al. [82], Zhang et al. [83]
SAE	GSR	Glutathionereductase	up	Carolan et al. [78], Hackett et al. [79]
SAE/LAE	ADH7	Alcoholdehydrogenase 7, mu or sigmapolypeptide	up	Carolan et al. [78], Hackett et al. [79], Harvey et al. [80], Turetz et al. [81], Spira et al. [82]
SAE/LAE	AKR1B1	aldo-keto reductasefamily 1, memberB1	up	Carolan et al. [78], Hackett et al. [79], Harvey et al. [80], Spira et al. [82], Zhang et al. [83]
SAE/LAE	AKR1C	aldo-keto reductasefamily 1, memberC	up	Carolan et al. [78], Hackett et al. [79], Harvey et al. [80], Turetz et al. [81], Spira et al. [82], Zhang et al. [83]
SAE	TXNRD1	Thioredoxinreductase 1	up	Carolan et al. [78], Hackett et al. [79], Spira et al. [82]
SAE	PGD	Phosphogluconatedehydrogenase	up	Carolan et al. [78], Hackett et al. [79]
SAE	TALDO1	transaldolase 1	up	Carolan et al. [78], Hackett et al. [79]
SAE	CYP1B1	cytochrome P450, family 1, subfamilyB, polypeptide1	up	Carolan et al. [78], Hackett et al. [79], Harvey et al. [80], Spira et al. [82], Zhang et al. [83]
SAE/LAE	CX3CLI	Chemokine(C-X3-C motif)ligand 1	down	Harvey et al. [80], Turetz et al. [81], Spira et al. [82]
SAE/LAE	ALDH3A1	Aldehydedehydrogenase 3family, memberA1	up	Harvey et al. [80], Turetz et al. [81], Spira et al. [82]
SAE/LAE	NQO1	NAD(P)Hdehydrogenase, quinone 1	up	Harvey et al. [80], Spira et al. [82], Zhang et al. [83]
LAE	SLIT	slit homolog (Drosophila)	up	Turetz et al. [81], Spira et al. [82]
SAE	UCHL1	Ubiquitincarboxylterminal, esteraseL1	down	Harvey et al. [80], Spira et al. [82]

^a^HS: healthy smokers, ^b^NS: nonsmokers

^c^SAE: small airway epithelium, ^d^LAE: large airway epithelium

Smoking induced epigenetic changes with corresponding modulation of gene expression are demonstrated both in airway epithelium and alveolar macrophages [90, 91]. Buro-Auriemma with his team [90] identified 204 unique genes differentially methylated in the small airway epithelium DNA of smokers compared with nonsmokers. Cigarette smoking is also associated with genome wide changes inpulmonary macrophage DNA methylation, in particular at the aryl hydrocarbon receptor repressor (AHRR), a known tumor suppressor that may be critical in moderating AHR role in oncogenesisand altered immune function [91].

## Structural changes

Since 1957, numerous studies have proved the structural changes of airway epithelium brought by smoking [92]. An increase in thickness of the epithelium with an elevation in size and number of goblet cells, a decrease in the length ofcilia, loss of cilia and occurrence of cells with atypical nuclei were revealed in both tracheal and bronchial epithelium of smokers compared with nonsmokers [92–94]. Besides, the percentages of individuals exhibiting precancerouslesions including basal cell hyperplasia and squamous metaplasia, increased with the habit of cigarette smoking [93, 95]. Robust structural changes in airway epithelial mitochondria induced by cigarette smoke were detected, such as fragmentation, branching and quantity of cristae [96]. While the changes as a consequence of tobacco mentioned above could be reversible. Bertram and Rogers [97] demonstrated that the structural recovery occurred in bronchial epithelium in people who stopped smoking for over two years. Healthy smokers’ cilia was much shorter than non-smokers’ which may affect the mucociliary clearance [84]. The reticular basement membrane of both smokers and individuals with COPD showed fragmentation and splitting [98]. More vessels in the reticular basement membrane and fewer vessels in the lamina propria were also found in current smokers compared to healthy nonsmokers [98], suggesting airway remodelling in smokers.

## Pulmonary dysfunction

Smoking is regarded as the major contribution to pulmonary dysfunction, and this deterioration of lung function in smokers is far in excess of that predicted by age [99]. The ventilation of the upper zones of the lungs was significantly less than that of the lower zones in smokers, suggesting the upper zone abnormalities found in the group of smokers were consistent with the development of early emphysema [100]. Although, parameters of pulmonary function test of healthy smokers are within the normal range, some abnormalities are detected by pulmonologiosts when compared with lifelong nonsmokers. Reduced forced expiratory volume in one second(FEV1), peak expiratory flow(PEF) and the ratio of FEV1 to forced vital capacity(FVC, FEV1/FVC), decreased diffusing capacity for CO and forced expiratory flows at high lung volume, increase in total lung capacity(TLC), the ratio of residual volume(RV) to TLC (RV/TLC) and the ratio of functional residual capacity(FRC) to TLC(FRC/TLC) were demonstrated in smokers [101–104]. Forced expiratory time for the last 0.5 l of the forced vital capacity was significantly higher in the heavy smokers (those who had smoked a lifetime total of more than 10,000 cigarettes) than the nonsmokers [102]. A linear association between smoking years and reduced level of FEV1 and FVC was reported [105] and the decline in FEV1 can also be detected in teenage smokers [106]. Moreover, smoking cessation not only stopped the smoking-induced fast decline in lung function, but even led to some reversal toward nonsmoking values [107]. Frette with his team [108] found that smokers who quit before age 40 had an age- and height-adjusted FEV1 that did not differ from that of never smokers in either men or women. In summary, these findings confirm the deleterious effect on lung function of smokers whose spirometry values are within normal range and prove a beneficial effect of quitting smoking at an early age.

## Systemic effects

### Systemic inflammation

Numerous studies have shown significantly increased level of white blood cell [109–111], TNF-α [26, 112] and C-reactive protein [113, 114] in serum of asymptomatic smokers with normal lung function, providing direct evidence of systemic inflammation in smokers. However, most of researches found no differences in the subsets of T-lymphocytes which mediate abnormal intrapulmonary inflammation and have been identified as a key component in the development and the progression of COPD in peripheral blood [29, 109, 115]. But, Miller et al. [116] detected the decreased level of CD4^+^T lymphocytes and increased level of CD8^+^T lymphocytes in peripheral blood from heavy smokers which may need further studies.

### Oxidative damage

Many researches have confirmed the systemic oxidative damage and overall decrease in antioxidant activity in smokers as compared with nonsmokers. Antwerpen et al. [117] demonstrated that smoking was associated with significantly increased phagocyte-derived ROS-generation. The mean plasma malondialdehyde(MDA) level, a parameter of lipid peroxidation caused by the oxidants, was higher both in healthy smokers and smokers with COPD than in healthy nonsmokers [118]. Besides, after 4 week smoking cessation,significant decreases in MDA were detected [119]. Significantly lower CuZnSOD and Se-GSH-Px activities have been reported both in teenage and adult smokers than non-smokers [120, 121]. Concentrations of serum antioxidants, such as folate, vitamin C and vitamin E, have been proved to be lower in chronic smokers [122, 123], confirming again smoking induced damage to the oxidant defense system.

### Endothelial dysfunction

Oxidative damage brought by smoking is closely related to endothelial dysfunction. Guthikonda with his colleagues [124] demonstrated xanthine oxidase contributes importantly to endothelial dysfunction caused by cigarette smoking. Hirai et al. [125] found that the impaired endothelial dysfunction in smokers could be improved by the antioxidant, vitamin C. In fact, lots of studies have proved endothelial dysfunction measured by flow-mediated dilation (FMD) in healthy smokers [126, 127]. Moreover, Mendes with his team [128] found that impaired airway vascular endothelial function nmight precede endothelial dysfunction of other areas in healthy smokers. Celermajer et al. [126] demonstrated an inverse relation of FMD and lifetime cigarette dose smoked and former smokers had higher FMD values than current smokers, indicating potentially reversible smoking induced endothelial dysfunction.

Reduced number of circulating endothelial progenitor cells (EPCs) is reported [129]. The number of EPCs is also indicated to be correlated with endothelial function as measured by FMD [130]. Furthermore, Michaud’s study [131] demonstrated the impairment of EPC differentiation and functional activities brought by smoking and this impairment might be associated with lower serum antioxidant levels. A recent study also identifies that epigenetic regulation of DNA damage and senescence are closely related to the endothelial progenitors’ dysfunction in both smokers and COPD patients [132]. While, smoking induced effect on circulating EPCs was reversible. Kondo et al. [133] observed that the recovery of EPC levels was greater in light smokers than in heavy smokers, suggesting the significance of smoking cessation.

### Effects of cardiovascular, nervous-mental, endocrine and reproductive system

Compared with nonsmokers, smokers have significantly elevated risk factors for cardiovascular diseases. Although, healthy smokers’ heart rate, blood pressure and level of serum lipid and lipoprotein are in the normal range, increases in heart rate and blood pressure are detected as compared with nonsmokers [134, 135], they also have higher serum concentrations of cholesterol, triglycerides, very low density lipoprotein cholesterol, and low density lipoprotein cholesterol and lower serum concentrations of high density lipoprotein cholesterol and apolipoprotein AI [136]. Endothelial dysfunction and elevated level of white blood cell in healthy smokers mentioned above also contribute a lot to the onset of cardiovascular diseases [137] and become an independent risk factor for all atherosclerotic cardiovascular diseases [138].

Among the healthy smokers who don’t have any metal illnesses including alcohol or drug abuse/dependence with normal brain MRI results, abnormalities are still detected in their nervous-mental system. Hao with his team [139] found that neural function was less synchronized in the right inferior frontal cortex and more synchronized in the left superior parietal lobe in chronic smokers compared to nonsmokers, indicating lacking of control over reward-related behavior and smoking urges respectively. Significantly greater rate atrophy over 2-years than nonsmokers in multiple brain regions associated with the early stages of Alzheimer Disease were found in healthy, cognitively-intact elderly smokers [140]. Furthermore, chronic smokers were demonstrated to have a worse visual memory and poorer sleep quality compared with lifelong nonsmokers [141]. Jiménez-Ruiz C.A. et al. [142] found that 10.2% of healthy smokers had high dependence on nicotine evaluated by the Fagerstrom Test for Nicotine Dependence (FTND) which is a quantitative scale used commonly for the definition of nicotine dependence. Actually, tobacco dependence itself is not only a bad habit, but a chronic disease [143].

Attvall et al. [144] demonstrated that smoking could impair insulin action and lead to insulin resistance in healthy smokers even their blood glucose was normal. Smoking cessation improving insulin sensitivity in healthy middle-aged men were also reported [145]. Besides, detrimental effects on reproductive system brought by smoking are detected. Mostafa with his colleagues [146] found that smoking had negative effects on sperm motility, viability, DNA fragmentation, seminal zinc levels, and semen reactive oxygen species levels, even in healthy fertile smokers.

## Conclusion

The biochemical analysis of the induced sputum, expired breath condensate, bronchoalveolar lavage, biopsies of lung tissue and peripheral blood from healthy smokers are strongly enough to prove the exist of local and systemic inflammation. Genetic alterations detected in the lung tissue of healthy smokers may contribute to smoking-related susceptibility to lung diseases, such as emphysema and lung cancer. The structural changes in respiratory system, as well as the decline in lung function as compared with nonsmokers demonstrate again smoking induced negative effects on healthy smokers which accelerate the onset of respiratory disorders. Therefore, healthy smokers who are normal with spirometry, radiographic images and routine physical exam are not really healthy. Smoking cessation as an early intervention may lead to some reversal toward the better health of lifelong nonsmokers.

## Abbreviations

BAL: Bronchoalveolar lavage
COPD: Chronic obstructive pulmonary disease
EBC: Expired breath condensate
EPCs: Endothelial progenitor cells
FEV1: Forced expiratory volume in one second
FMD: Flow-mediated dilation
FRC: Functional residual capacity
FVC: Forced vital capacity
HS: Healthy smokers
LAE: Large airway epithelium
MCP-1: Monocyte chemoattractant protein-1
MDA: Malondialdehyde
MMP: Matrix metalloproteinase
NF-kB: Nuclear factor-kB
NS: Nonsmokers
PEF: Peak expiratory flow
RV: Residual volume
SAE: Small airway epithelium
TLC: Total lung capacity
TLR: The toll-like receptor
TNF-α: Tumor necrosis factor alpha

## References

[1] Lee J, Taneja V, Vassallo R (2012). Cigarette smoking and inflammation: cellular and molecular mechanisms. J Dent Res.

[2] Teo KK, Ounpuu S, Hawken S, Pandey MR, Valentin V, Hunt D, Diaz R, Rashed W, Freeman R, Jiang L, Zhang X, Yusuf S (2006). Tobacco use and risk of myocardial infarction in 52 countries in the INTERHEART study: a case–control study. Lancet.

[3] Jindal SK, Aggarwal AN, Chaudhry K, Chhabra SK, D’Souza GA, Gupta D, Katiyar SK, Kumar R, Shah B, Vijayan VK (2006). A multicentric study on epidemiology of chronic obstructive pulmonary disease and its relationship with tobacco smoking and environmental tobacco smoke exposure. Indian J Chest Dis Allied Sci.

[4] Lubin JH, Blot WJ, Berrino F, Flamant R, Gillis CR, Kunze M, Schmahl D, Visco G (1984). Modifying risk of developing lung cancer by changing habits of cigarette smoking. Br Med J (Clin Res Ed).

[5] Wang R, Wang G, Ricard MJ, Ferris B, Strulovici-Barel Y, Salit J, Hackett NR, Gudas LJ, Crystal RG (2010). Smoking-induced upregulation of AKR1B10 expression in the airway epithelium of healthy individuals. Chest.

[6] Swan GE, Hodgkin JE, Roby T, Mittman C, Jacobo N, Peters J (1992). Reversibility of airways injury over a 12-month period following smoking cessation. Chest.

[7] Besaratinia A, Maas LM, Van Breda SG, Curfs DM, Kleinjans JC, Wouters EF, Van Schooten FJ (2000). Applicability of induced sputum for molecular dosimetry of exposure to inhalatory carcinogens: 32P-postlabeling of lipophilic DNA adducts in smokers and nonsmokers. Cancer Epidemiol Biomarkers Prev.

[8] Mazur W, Toljamo T, Ohlmeier S, Vuopala K, Nieminen P, Kobayashi H, Kinnula VL (2011). Elevation of surfactant protein A in plasma and sputum in cigarette smokers. Eur Respir J.

[9] Rufino R, Costa CH, Souza HS, Madi K, Silva JR (2007). Induced sputum and peripheral blood cell profile in chronic obstructive pulmonary disease. J Bras Pneumol.

[10] Domagala-Kulawik J, Maskey-Warzechowska M, Hermanowicz-Salamon J, Chazan R (2006). Expression of macrophage surface markers in induced sputum of patients with chronic obstructive pulmonary disease. J Physiol Pharmacol.

[11] Skold CM, Lundahl J, Hallden G, Hallgren M, Eklund A (1996). Chronic smoke exposure alters the phenotype pattern and the metabolic response in human alveolar macrophages. Clin Exp Immunol.

[12] Le-Barillec K, Si-Tahar M, Balloy V, Chignard M (1999). Proteolysis of monocyte CD14 by human leukocyte elastase inhibits lipopolysaccharide-mediated cell activation. J Clin Invest.

[13] Bouloukaki I, Tsoumakidou M, Vardavas CI, Mitrouska I, Koutala E, Siafakas NM, Schiza SE, Tzanakis N (2009). Maintained smoking cessation for 6 months equilibrates the percentage of sputum CD8+ lymphocyte cells with that of nonsmokers. Mediators Inflamm.

[14] Takanashi S, Hasegawa Y, Kanehira Y, Yamamoto K, Fujimoto K, Satoh K, Okamura K (1999). Interleukin-10 level in sputum is reduced in bronchial asthma, COPD and in smokers. Eur Respir J.

[15] Costa C, Rufino R, Traves SL, Lapa ESJR, Barnes PJ, Donnelly LE (2008). CXCR3 and CCR5 chemokines in induced sputum from patients with COPD. Chest.

[16] Wang F, He B (2012). CCR1 and CCR5 expression on inflammatory cells is related to cigarette smoking and chronic obstructive pulmonary disease severity. Chin Med J (Engl).

[17] Hacievliyagil SS, Mutlu LC, Temel I (2013). Airway inflammatory markers in chronic obstructive pulmonary disease patients and healthy smokers. Niger J Clin Pract.

[18] Chung KF (2001). Cytokines in chronic obstructive pulmonary disease. Eur Respir J Suppl.

[19] Kostikas K, Papatheodorou G, Ganas K, Psathakis K, Panagou P, Loukides S (2002). pH in expired breath condensate of patients with inflammatory airway diseases. Am J Respir Crit Care Med.

[20] MacNee W, Rennard SI, Hunt JF, Edwards LD, Miller BE, Locantore NW, Tal-Singer R (2011). Evaluation of exhaled breath condensate pH as a biomarker for COPD. Respir Med.

[21] Koczulla AR, Noeske S, Herr C, Jorres RA, Rommelt H, Vogelmeier C, Bals R (2010). Acute and chronic effects of smoking on inflammation markers in exhaled breath condensate in current smokers. Respiration.

[22] Nicola ML, Carvalho HB, Yoshida CT, Anjos FM, Nakao M, Santos Ude P, Cardozo KH, Carvalho VM, Pinto E, Farsky SH, Saldiva PH, Rubin BK, Nakagawa NK (2014). Young “healthy” smokers have functional and inflammatory changes in the nasal and the lower airways. Chest.

[23] Carpagnano GE, Kharitonov SA, Foschino-Barbaro MP, Resta O, Gramiccioni E, Barnes PJ (2003). Increased inflammatory markers in the exhaled breath condensate of cigarette smokers. Eur Respir J.

[24] Garey KW, Neuhauser MM, Robbins RA, Danziger LH, Rubinstein I (2004). Markers of inflammation in exhaled breath condensate of young healthy smokers. Chest.

[25] Corhay JL, Hemelaers L, Henket M, Sele J, Louis R (2007). Granulocyte chemotactic activity in exhaled breath condensate of healthy subjects and patients with COPD. Chest.

[26] Diez-Pina JM, Fernandez-Acenero MJ, Llorente-Alonso MJ, Diaz-Lobato S, Mayoralas S, Florez A (2009). Tumor necrosis factor alpha as a marker of systemic and local inflammation in “healthy” smokers. Int J Gen Med.

[27] Montuschi P, Collins JV, Ciabattoni G, Lazzeri N, Corradi M, Kharitonov SA, Barnes PJ (2000). Exhaled 8-isoprostane as an in vivo biomarker of lung oxidative stress in patients with COPD and healthy smokers. Am J Respir Crit Care Med.

[28] Reynolds HY, Newball HH (1974). Analysis of proteins and respiratory cells obtained from human lungs by bronchial lavage. J Lab Clin Med.

[29] Wallace JM, Oishi JS, Barbers RG, Simmons MS, Tashkin DP (1994). Lymphocytic subpopulation profiles in bronchoalveolar lavage fluid and peripheral blood from tobacco and marijuana smokers. Chest.

[30] Rutgers SR, Timens W, Kaufmann HF, van der Mark TW, Koeter GH, Postma DS (2000). Comparison of induced sputum with bronchial wash, bronchoalveolar lavage and bronchial biopsies in COPD. Eur Respir J.

[31] Erjefalt JS, Uller L, Malm-Erjefalt M, Persson CG (2004). Rapid and efficient clearance of airway tissue granulocytes through transepithelial migration. Thorax.

[32] Yu MQ, Liu XS, Wang JM, Xu YJ (2013). CD8(+) Tc-lymphocytes immunodeviation in peripheral blood and airway from patients of chronic obstructive pulmonary disease and changes after short-term smoking cessation. Chin Med J (Engl).

[33] Saetta M, Di Stefano A, Turato G, Facchini FM, Corbino L, Mapp CE, Maestrelli P, Ciaccia A, Fabbri LM (1998). CD8+ T-lymphocytes in peripheral airways of smokers with chronic obstructive pulmonary disease. Am J Respir Crit Care Med.

[34] Mosmann TR, Li L, Sad S (1997). Functions of CD8 T-cell subsets secreting different cytokine patterns. Semin Immunol.

[35] Kuschner WG, D’Alessandro A, Wong H, Blanc PD (1996). Dose-dependent cigarette smoking-related inflammatory responses in healthy adults. Eur Respir J.

[36] Blomberg A, Mudway I, Svensson M, Hagenbjork-Gustafsson A, Thomasson L, Helleday R, Dumont X, Forsberg B, Nordberg G, Bernard A (2003). Clara cell protein as a biomarker for ozone-induced lung injury in humans. Eur Respir J.

[37] Shijubo N, Itoh Y, Yamaguchi T, Shibuya Y, Morita Y, Hirasawa M, Okutani R, Kawai T, Abe S (1997). Serum and BAL Clara cell 10 kDa protein (CC10) levels and CC10-positive bronchiolar cells are decreased in smokers. Eur Respir J.

[38] Lim S, Roche N, Oliver BG, Mattos W, Barnes PJ, Chung KF (2000). Balance of matrix metalloprotease-9 and tissue inhibitor of metalloprotease-1 from alveolar macrophages in cigarette smokers. Regulation by interleukin-10. Am J Respir Crit Care Med.

[39] Babusyte A, Stravinskaite K, Jeroch J, Lotvall J, Sakalauskas R, Sitkauskiene B (2007). Patterns of airway inflammation and MMP-12 expression in smokers and ex-smokers with COPD. Respir Res.

[40] McCormack FX, Whitsett JA (2002). The pulmonary collectins, SP-A and SP-D, orchestrate innate immunity in the lung. J Clin Invest.

[41] Honda Y, Takahashi H, Kuroki Y, Akino T, Abe S (1996). Decreased contents of surfactant proteins A and D in BAL fluids of healthy smokers. Chest.

[42] Pacht ER, Kaseki H, Mohammed JR, Cornwell DG, Davis WB (1986). Deficiency of vitamin E in the alveolar fluid of cigarette smokers. Influence on alveolar macrophage cytotoxicity. J Clin Invest.

[43] Pons AR, Sauleda J, Noguera A, Pons J, Barcelo B, Fuster A, Agusti AG (2005). Decreased macrophage release of TGF-beta and TIMP-1 in chronic obstructive pulmonary disease. Eur Respir J.

[44] Harris JO, Swenson EW, Johnson JE (1970). Human alveolar macrophages: comparison of phagocytic ability, glucose utilization, and ultrastructure in smokers and nonsmokers. J Clin Invest.

[45] Pratt SA, Smith MH, Ladman AJ, Finley TN (1971). The ultrastructure of alveolar macrophages from human cigarette smokers and nonsmokers. Lab Invest.

[46] Harris JO, Olsen GN, Castle JR, Maloney AS (1975). Comparison of proteolytic enzyme activity in pulmonary alveolar macrophages and blood leukocytes in smokers and nonsmokers. Am Rev Respir Dis.

[47] Hinman LM, Stevens CA, Matthay RA, Gee JB (1980). Elastase and lysozyme activities in human alveolar macrophages. Effects of cigarette smoking. Am Rev Respir Dis.

[48] Fisher GL, McNeill KL, Finch GL, Wilson FD, Golde DW (1982). Functional evaluation of lung macrophages from cigarette smokers and nonsmokers. J Reticuloendothel Soc.

[49] Nagai S, Takeuchi M, Watanabe K, Aung H, Izumi T (1988). Smoking and interleukin-1 activity released from human alveolar macrophages in healthy subjects. Chest.

[50] Ohta T, Yamashita N, Maruyama M, Sugiyama E, Kobayashi M (1998). Cigarette smoking decreases interleukin-8 secretion by human alveolar macrophages. Respir Med.

[51] Soliman DM, Twigg HL (1992). Cigarette smoking decreases bioactive interleukin-6 secretion by alveolar macrophages. Am J Physiol.

[52] Yamaguchi E, Itoh A, Furuya K, Miyamoto H, Abe S, Kawakami Y (1993). Release of tumor necrosis factor-alpha from human alveolar macrophages is decreased in smokers. Chest.

[53] Hoidal JR, Fox RB, LeMarbre PA, Takiff HE, Repine JE (1980). Oxidative metabolism of alveolar macrophages from young asymptomatic cigarette smokers. Increased superoxide anion release and its potential consequences. Chest.

[54] Hubbard RC, Ogushi F, Fells GA, Cantin AM, Jallat S, Courtney M, Crystal RG (1987). Oxidants spontaneously released by alveolar macrophages of cigarette smokers can inactivate the active site of alpha 1-antitrypsin, rendering it ineffective as an inhibitor of neutrophil elastase. J Clin Invest.

[55] Schaberg T, Lauer C, Lode H, Fischer J, Haller H (1992). Increased number of alveolar macrophages expressing adhesion molecules of the leukocyte adhesion molecule family in smoking subjects. Association with cell-binding ability and superoxide anion production. Am Rev Respir Dis.

[56] Shaykhiev R, Krause A, Salit J, Strulovici-Barel Y, Harvey BG, O’Connor TP, Crystal RG (2009). Smoking-dependent reprogramming of alveolar macrophage polarization: implication for pathogenesis of chronic obstructive pulmonary disease. J Immunol.

[57] Roth MD, Arora A, Barsky SH, Kleerup EC, Simmons M, Tashkin DP (1998). Airway inflammation in young marijuana and tobacco smokers. Am J Respir Crit Care Med.

[58] Lams BE, Sousa AR, Rees PJ, Lee TH (1998). Immunopathology of the small-airway submucosa in smokers with and without chronic obstructive pulmonary disease. Am J Respir Crit Care Med.

[59] Ricciardolo FL, Caramori G, Ito K, Capelli A, Brun P, Abatangelo G, Papi A, Chung KF, Adcock I, Barnes PJ, Donner CF, Rossi A, Di Stefano A (2005). Nitrosative stress in the bronchial mucosa of severe chronic obstructive pulmonary disease. J Allergy Clin Immunol.

[60] Di Stefano A, Caramori G, Gnemmi I, Contoli M, Vicari C, Capelli A, Magno F, D’Anna SE, Zanini A, Brun P, Casolari P, Chung KF, Barnes PJ, Papi A, Adcock I, Balbi B (2009). T helper type 17-related cytokine expression is increased in the bronchial mucosa of stable chronic obstructive pulmonary disease patients. Clin Exp Immunol.

[61] Isajevs S, Taivans I, Svirina D, Strazda G, Kopeika U (2011). Patterns of inflammatory responses in large and small airways in smokers with and without chronic obstructive pulmonary disease. Respiration.

[62] Rahman I, Marwick J, Kirkham P (2004). Redox modulation of chromatin remodeling: impact on histone acetylation and deacetylation, NF-kappaB and pro-inflammatory gene expression. Biochem Pharmacol.

[63] Di Stefano A, Caramori G, Oates T, Capelli A, Lusuardi M, Gnemmi I, Ioli F, Chung KF, Donner CF, Barnes PJ, Adcock IM (2002). Increased expression of nuclear factor-kappaB in bronchial biopsies from smokers and patients with COPD. Eur Respir J.

[64] Raghuwanshi SK, Su Y, Singh V, Haynes K, Richmond A, Richardson RM (2012). The chemokine receptors CXCR1 and CXCR2 couple to distinct G protein-coupled receptor kinases to mediate and regulate leukocyte functions. J Immunol.

[65] Di Stefano A, Caramori G, Gnemmi I, Contoli M, Bristot L, Capelli A, Ricciardolo FL, Magno F, D’Anna SE, Zanini A, Carbone M, Sabatini F, Usai C, Brun P, Chung KF, Barnes PJ, Papi A, Adcock IM, Balbi B (2009). Association of increased CCL5 and CXCL7 chemokine expression with neutrophil activation in severe stable COPD. Thorax.

[66] Wang R, Ahmed J, Wang G, Hassan I, Strulovici-Barel Y, Salit J, Mezey JG, Crystal RG (2012). Airway epithelial expression of TLR5 is downregulated in healthy smokers and smokers with chronic obstructive pulmonary disease. J Immunol.

[67] Letran SE, Lee SJ, Atif SM, Flores-Langarica A, Uematsu S, Akira S, Cunningham AF, McSorley SJ (2011). TLR5-deficient mice lack basal inflammatory and metabolic defects but exhibit impaired CD4 T cell responses to a flagellated pathogen. J Immunol.

[68] Woodruff PG, Koth LL, Yang YH, Rodriguez MW, Favoreto S, Dolganov GM, Paquet AC, Erle DJ (2005). A distinctive alveolar macrophage activation state induced by cigarette smoking. Am J Respir Crit Care Med.

[69] Graff JW, Powers LS, Dickson AM, Kim J, Reisetter AC, Hassan IH, Kremens K, Gross TJ, Wilson ME, Monick MM (2012). Cigarette smoking decreases global microRNA expression in human alveolar macrophages. PLoS One.

[70] Philibert RA, Sears RA, Powers LS, Nash E, Bair T, Gerke AK, Hassan I, Thomas CP, Gross TJ, Monick MM (2012). Coordinated DNA methylation and gene expression changes in smoker alveolar macrophages: specific effects on VEGF receptor 1 expression. J Leukoc Biol.

[71] Heguy A, O’Connor TP, Luettich K, Worgall S, Cieciuch A, Harvey BG, Hackett NR, Crystal RG (2006). Gene expression profiling of human alveolar macrophages of phenotypically normal smokers and nonsmokers reveals a previously unrecognized subset of genes modulated by cigarette smoking. J Mol Med (Berl).

[72] Rose RM, Kobzik L, Filderman AE, Vermeulen MW, Dushay K, Donahue RE (1992). Characterization of colony stimulating factor activity in the human respiratory tract. Comparison of healthy smokers and nonsmokers. Am Rev Respir Dis.

[73] Hautamaki RD, Kobayashi DK, Senior RM, Shapiro SD (1997). Requirement for macrophage elastase for cigarette smoke-induced emphysema in mice. Science.

[74] Qu P, Du H, Wang X, Yan C (2009). Matrix metalloproteinase 12 overexpression in lung epithelial cells plays a key role in emphysema to lung bronchioalveolar adenocarcinoma transition. Cancer Res.

[75] Spivack SD, Hurteau GJ, Reilly AA, Aldous KM, Ding X, Kaminsky LS (2001). CYP1B1 expression in human lung. Drug Metab Dispos.

[76] Lao X, Qin X, Peng Q, Chen Z, Lu Y, Liu Y, Li S. Association of CYP1B1 Leu432Val Polymorphism and Lung Cancer Risk: An Updated Meta-Analysis. Lung. 2014. 10.1007/s00408-014-9618-1 24989113

[77] Tam A, Wadsworth S, Dorscheid D, Man SF, Sin DD (2011). The airway epithelium: more than just a structural barrier. Ther Adv Respir Dis.

[78] Carolan BJ, Harvey BG, Hackett NR, O’Connor TP, Cassano PA, Crystal RG (2009). Disparate oxidant gene expression of airway epithelium compared to alveolar macrophages in smokers. Respir Res.

[79] Hackett NR, Heguy A, Harvey BG, O’Connor TP, Luettich K, Flieder DB, Kaplan R, Crystal RG (2003). Variability of antioxidant-related gene expression in the airway epithelium of cigarette smokers. Am J Respir Cell Mol Biol.

[80] Harvey BG, Heguy A, Leopold PL, Carolan BJ, Ferris B, Crystal RG (2007). Modification of gene expression of the small airway epithelium in response to cigarette smoking. J Mol Med (Berl).

[81] Turetz ML, O’Connor TP, Tilley AE, Strulovici-Barel Y, Salit J, Dang D, Teater M, Mezey J, Clark AG, Crystal RG (2009). Trachea epithelium as a “canary” for cigarette smoking-induced biologic phenotype of the small airway epithelium. Clin Transl Sci.

[82] Spira A, Beane J, Shah V, Liu G, Schembri F, Yang X, Palma J, Brody JS (2004). Effects of cigarette smoke on the human airway epithelial cell transcriptome. Proc Natl Acad Sci U S A.

[83] Zhang L, Lee JJ, Tang H, Fan YH, Xiao L, Ren H, Kurie J, Morice RC, Hong WK, Mao L (2008). Impact of smoking cessation on global gene expression in the bronchial epithelium of chronic smokers. Cancer Prev Res (Phila).

[84] Leopold PL, O’Mahony MJ, Lian XJ, Tilley AE, Harvey BG, Crystal RG (2009). Smoking is associated with shortened airway cilia. PLoS One.

[85] Hessel J, Heldrich J, Fuller J, Staudt MR, Radisch S, Hollmann C, Harvey BG, Kaner RJ, Salit J, Yee-Levin J, Sridhar S, Pillai S, Hilton H, Wolff G, Bitter H, Visvanathan S, Fine J, Stevenson CS, Crystal RG, Tilley AE (2014). Intraflagellar transport gene expression associated with short cilia in smoking and COPD. PLoS One.

[86] Wang G, Xu Z, Wang R, Al-Hijji M, Salit J, Strulovici-Barel Y, Tilley AE, Mezey JG, Crystal RG (2012). Genes associated with MUC5AC expression in small airway epithelium of human smokers and non-smokers. BMC Med Genomics.

[87] Shaykhiev R, Otaki F, Bonsu P, Dang DT, Teater M, Strulovici-Barel Y, Salit J, Harvey BG, Crystal RG (2011). Cigarette smoking reprograms apical junctional complex molecular architecture in the human airway epithelium in vivo. Cell Mol Life Sci.

[88] Carolan BJ, Heguy A, Harvey BG, Leopold PL, Ferris B, Crystal RG (2006). Up-regulation of expression of the ubiquitin carboxyl-terminal hydrolase L1 gene in human airway epithelium of cigarette smokers. Cancer Res.

[89] Tilley AE, Harvey BG, Heguy A, Hackett NR, Wang R, O’Connor TP, Crystal RG (2009). Down-regulation of the notch pathway in human airway epithelium in association with smoking and chronic obstructive pulmonary disease. Am J Respir Crit Care Med.

[90] Buro-Auriemma LJ, Salit J, Hackett NR, Walters MS, Strulovici-Barel Y, Staudt MR, Fuller J, Mahmoud M, Stevenson CS, Hilton H, Ho MW, Crystal RG (2013). Cigarette smoking induces small airway epithelial epigenetic changes with corresponding modulation of gene expression. Hum Mol Genet.

[91] Monick MM, Beach SR, Plume J, Sears R, Gerrard M, Brody GH, Philibert RA (2012). Coordinated changes in AHRR methylation in lymphoblasts and pulmonary macrophages from smokers. Am J Med Genet B Neuropsychiatr Genet.

[92] Chang SC (1957). Microscopic properties of whole mounts and sections of human bronchial epithelium of smokers and nonsmokers. Cancer.

[93] Ide G, Suntzeff V, Cowdry EV (1959). A comparison of the histopathology of tracheal and bronchial epithelium of smokers and nonsmokers. Cancer.

[94] Auerbach O, Hammond EC, Garfinkel L (1979). Changes in bronchial epithelium in relation to cigarette smoking, 1955–1960 vs. 1970–1977.. N Engl J Med.

[95] Peters EJ, Morice R, Benner SE, Lippman S, Lukeman J, Lee JS, Ro JY, Hong WK (1993). Squamous metaplasia of the bronchial mucosa and its relationship to smoking. Chest.

[96] Hoffmann RF, Zarrintan S, Brandenburg SM, Kol A, de Bruin HG, Jafari S, Dijk F, Kalicharan D, Kelders M, Gosker HR, Ten Hacken NH, van der Want JJ, van Oosterhout AJ, Heijink IH (2013). Prolonged cigarette smoke exposure alters mitochondrial structure and function in airway epithelial cells. Respir Res.

[97] Bertram JF, Rogers AW (1981). Recovery of bronchial epithelium on stopping smoking. Br Med J (Clin Res Ed).

[98] Soltani A, Reid DW, Sohal SS, Wood-Baker R, Weston S, Muller HK, Walters EH (2010). Basement membrane and vascular remodelling in smokers and chronic obstructive pulmonary disease: a cross-sectional study. Respir Res.

[99] Corbin RP, Loveland M, Martin RR, Macklem PT (1979). A four-year follow-up study of lung mechanics in smokers. Am Rev Respir Dis.

[100] Seaton D, Ogilvie CM (1978). Regional lung function in asymptomatic cigarette smokers. Am Rev Respir Dis.

[101] Seppanen A (1977). Comparison of different kinds of tests in the evaluation of lung function among healthy smokers and nonsmokers. Ann Clin Res.

[102] Walter S, Nancy NR, Collier CR (1979). Changes in the forced expiratory spirogram in young male smokers. Am Rev Respir Dis.

[103] Barter SJ, Cunningham DA, Lavender JP, Gibellino F, Connellan SJ, Pride NB (1985). Abnormal ventilation scans in middle-aged smokers. Comparison with tests of overall lung function. Am Rev Respir Dis.

[104] Bosse R, Sparrow D, Garvey AJ, Costa PT, Weiss ST, Rowe JW (1980). Cigarette smoking, aging, and decline in pulmonary function: A longitudinal study. Arch Environ Health.

[105] Xu X, Li B, Wang L (1994). Gender difference in smoking effects on adult pulmonary function. Eur Respir J.

[106] Lee SK, Park JW, Kim KH, Jung JH (2013). An Analysis of the Thickness of Abdominal Muscles during Forceful Expiration and Pulmonary Function in Teenage Smokers and Nonsmokers. J Phys Ther Sci.

[107] Nemery B, Moavero NE, Brasseur L, Stanescu DC (1982). Changes in lung function after smoking cessation: an assessment from a cross-sectional survey. Am Rev Respir Dis.

[108] Frette C, Barrett-Connor E, Clausen JL (1996). Effect of active and passive smoking on ventilatory function in elderly men and women. Am J Epidemiol.

[109] Costabel U, Bross KJ, Reuter C, Ruhle KH, Matthys H (1986). Alterations in immunoregulatory T-cell subsets in cigarette smokers. A phenotypic analysis of bronchoalveolar and blood lymphocytes. Chest.

[110] Ortlepp JR, Metrikat J, Vesper K, Mevissen V, Schmitz F, Albrecht M, Maya-Pelzer P, Hanrath P, Weber C, Zerres K, Hoffmann R (2003). The interleukin-6 promoter polymorphism is associated with elevated leukocyte, lymphocyte, and monocyte counts and reduced physical fitness in young healthy smokers. J Mol Med (Berl).

[111] Fernandez JA, Prats JM, Artero JV, Mora AC, Farinas AV, Espinal A, Mendez JA (2012). Systemic inflammation in 222.841 healthy employed smokers and nonsmokers: white blood cell count and relationship to spirometry. Tob Induc Dis.

[112] Petrescu F, Voican SC, Silosi I (2010). Tumor necrosis factor-alpha serum levels in healthy smokers and nonsmokers. Int J Chron Obstruct Pulmon Dis.

[113] Alyan O, Kacmaz F, Ozdemir O, Karahan Z, Taskesen T, Iyem H, Alan S, Karadede A, Ilkay E (2008). High levels of high-sensitivity C-reactive protein and impaired autonomic activity in smokers. Turk Kardiyol Dern Ars.

[114] Dogan MV, Shields B, Cutrona C, Gao L, Gibbons FX, Simons R, Monick M, Brody GH, Tan K, Beach SR, Philibert RA (2014). The effect of smoking on DNA methylation of peripheral blood mononuclear cells from African American women. BMC Genomics.

[115] Mathai RT, Bhat S (2013). Peripheral blood T-cell populations in COPD, asymptomatic smokers and healthy Non-smokers in Indian subpopulation- a pilot study. J Clin Diagn Res.

[116] Miller LG, Goldstein G, Murphy M, Ginns LC (1982). Reversible alterations in immunoregulatory T cells in smoking. Analysis by monoclonal antibodies and flow cytometry. Chest.

[117] van Antwerpen VL, Theron AJ, Richards GA, Steenkamp KJ, van der Merwe CA, van der Walt R, Anderson R (1995). Vitamin E, pulmonary functions, and phagocyte-mediated oxidative stress in smokers and nonsmokers. Free Radic Biol Med.

[118] Ben Moussa S, Sfaxi I, Tabka Z, Ben Saad H, Rouatbi S (2014). Oxidative stress and lung function profiles of male smokers free from COPD compared to those with COPD: a case–control study. Libyan J Med.

[119] Erguder IB, Erguder T, Ozkan C, Bozkurt N, Soylu K, Devrim E, Durak I (2006). Short-term effects of smoking cessation on blood antioxidant parameters and paraoxonase activity in healthy asymptomatic long-term cigarette smokers. Inhal Toxicol.

[120] Kim SH, Kim JS, Shin HS, Keen CL (2003). Influence of smoking on markers of oxidative stress and serum mineral concentrations in teenage girls in Korea. Nutrition.

[121] Hulea SA, Olinescu R, Nita S, Crocnan D, Kummerow FA (1995). Cigarette smoking causes biochemical changes in blood that are suggestive of oxidative stress: a case–control study. J Environ Pathol Toxicol Oncol.

[122] Abou-Seif MA (1996). Blood antioxidant status and urine sulfate and thiocyanate levels in smokers. J Biochem Toxicol.

[123] Kim SH, Ensunsa JL, Zhu QY, Kim JS, Shin HS, Keen CL (2004). An 18-month follow-up study on the influence of smoking on blood antioxidant status of teenage girls in comparison with adult male smokers in Korea. Nutrition.

[124] Guthikonda S, Sinkey C, Barenz T, Haynes WG (2003). Xanthine oxidase inhibition reverses endothelial dysfunction in heavy smokers. Circulation.

[125] Hirai N, Kawano H, Hirashima O, Motoyama T, Moriyama Y, Sakamoto T, Kugiyama K, Ogawa H, Nakao K, Yasue H (2000). Insulin resistance and endothelial dysfunction in smokers: effects of vitamin C. Am J Physiol Heart Circ Physiol.

[126] Celermajer DS, Sorensen KE, Georgakopoulos D, Bull C, Thomas O, Robinson J, Deanfield JE (1993). Cigarette smoking is associated with dose-related and potentially reversible impairment of endothelium-dependent dilation in healthy young adults. Circulation.

[127] Yufu K, Takahashi N, Okada N, Shinohara T, Hara M, Saikawa T, Yoshimatsu H (2009). Influence of systolic blood pressure and cigarette smoking on endothelial function in young healthy people. Circ J.

[128] Mendes ES, Cancado JE, Rebolledo P, Arana J, Parker M, Gonzalez A, Hurwitz BE, Wanner A (2013). Airway vascular endothelial function in healthy smokers without systemic endothelial dysfunction. Chest.

[129] Heiss C, Amabile N, Lee AC, Real WM, Schick SF, Lao D, Wong ML, Jahn S, Angeli FS, Minasi P, Springer ML, Hammond SK, Glantz SA, Grossman W, Balmes JR, Yeghiazarians Y (2008). Brief secondhand smoke exposure depresses endothelial progenitor cells activity and endothelial function: sustained vascular injury and blunted nitric oxide production. J Am Coll Cardiol.

[130] Hill JM, Zalos G, Halcox JP, Schenke WH, Waclawiw MA, Quyyumi AA, Finkel T (2003). Circulating endothelial progenitor cells, vascular function, and cardiovascular risk. N Engl J Med.

[131] Michaud SE, Dussault S, Haddad P, Groleau J, Rivard A (2006). Circulating endothelial progenitor cells from healthy smokers exhibit impaired functional activities. Atherosclerosis.

[132] Paschalaki KE, Starke RD, Hu Y, Mercado N, Margariti A, Gorgoulis VG, Randi AM, Barnes PJ (2013). Dysfunction of endothelial progenitor cells from smokers and chronic obstructive pulmonary disease patients due to increased DNA damage and senescence. Stem Cells.

[133] Kondo T, Hayashi M, Takeshita K, Numaguchi Y, Kobayashi K, Iino S, Inden Y, Murohara T (2004). Smoking cessation rapidly increases circulating progenitor cells in peripheral blood in chronic smokers. Arterioscler Thromb Vasc Biol.

[134] Adan A, Sanchez-Turet M (1995). Smoking effects on diurnal variations of cardiovascular parameters. Int J Psychophysiol.

[135] Zevin S, Saunders S, Gourlay SG, Jacob P, Benowitz NL (2001). Cardiovascular effects of carbon monoxide and cigarette smoking. J Am Coll Cardiol.

[136] Craig WY, Palomaki GE, Haddow JE (1989). Cigarette smoking and serum lipid and lipoprotein concentrations: an analysis of published data. BMJ.

[137] Michael PR (2000). Cigarette smoking, endothelial injury and cardiovascular disease. Int J Exp Pathol.

[138] Jee SH, Park JY, Kim HS, Lee TY, Samet JM (2005). White blood cell count and risk for all-cause, cardiovascular, and cancer mortality in a cohort of Koreans. Am J Epidemiol.

[139] Tang J, Liao Y, Deng Q, Liu T, Chen X, Wang X, Xiang X, Chen H, Hao W (2012). Altered spontaneous activity in young chronic cigarette smokers revealed by regional homogeneity. Behav Brain Funct.

[140] Durazzo TC, Insel PS, Weiner MW (2012). Greater regional brain atrophy rate in healthy elderly subjects with a history of cigarette smoking. Alzheimers Dement.

[141] Liu JT, Lee IH, Wang CH, Chen KC, Lee CI, Yang YK (2013). Cigarette smoking might impair memory and sleep quality. J Formos Med Assoc.

[142] Jimenez-Ruiz CA, Masa F, Miravitlles M, Gabriel R, Viejo JL, Villasante C, Sobradillo V (2001). Smoking characteristics: differences in attitudes and dependence between healthy smokers and smokers with COPD. Chest.

[143] 2008 PHS Guideline Update Panel, Liaisons, and Staff (2008). Treating tobacco use and dependence: 2008 update U.S. Public Health Service Clinical Practice Guideline executive summary. Respir Care.

[144] Attvall S, Fowelin J, Lager I, Von Schenck H, Smith U (1993). Smoking induces insulin resistance--a potential link with the insulin resistance syndrome. J Intern Med.

[145] Eliasson B, Attvall S, Taskinen MR, Smith U (1997). Smoking cessation improves insulin sensitivity in healthy middle-aged men. Eur J Clin Invest.

[146] Taha EA, Ez-Aldin AM, Sayed SK, Ghandour NM, Mostafa T (2012). Effect of smoking on sperm vitality, DNA integrity, seminal oxidative stress, zinc in fertile men. Urology.

